# Vimentin Is at the Heart of Epithelial Mesenchymal Transition (EMT) Mediated Metastasis

**DOI:** 10.3390/cancers13194985

**Published:** 2021-10-05

**Authors:** Saima Usman, Naushin H. Waseem, Thuan Khanh Ngoc Nguyen, Sahar Mohsin, Ahmad Jamal, Muy-Teck Teh, Ahmad Waseem

**Affiliations:** 1 Centre for Oral Immunobiology and Regenerative Medicine, Institute of Dentistry, Barts and The London School of Medicine and Dentistry, Queen Mary University of London, Turner Str., London E1 2AT, UK; s.usman@qmul.ac.uk (S.U.); ntkngoc@web.de (T.K.N.N.); a.jamal@qmul.ac.uk (A.J.); m.t.teh@qmul.ac.uk (M.-T.T.); 2 UCL Institute of Ophthalmology, 11-43 Bath Str., London EC1V 9EL, UK; n.waseem@qmul.ac.uk; 3 Department of Anatomy, College of Medicine and Health Sciences, United Arab Emirates University, Al Ain 17666, United Arab Emirates; smohsin@uaeu.ac.ae

**Keywords:** cancer invasion, mesenchymal epithelial transition, cancer stem cells, epithelial tumours, amoeboid movement

## Abstract

**Simple Summary:**

Vimentin is an important filamentous protein providing structural and functional support to the cell. During initial stages of cancer development, vimentin concentration is very low, however, it increases when cancer starts to invade the surrounding areas. This review highlights the varied roles of vimentin in cancer growth and its spread to distant areas of the body. We have tried to explore the potential new areas of research related to the role of vimentin in cancer progression. We have also highlighted the reported damage to the vimentin gene in cancers, although how the damaged vimentin helps in cancer growth and spread is not known. We propose that latest technologies should be employed to medicinally target vimentin to reduce the cancer growth and its spread thereby helping to increase treatment outcomes and patients’ survival.

**Abstract:**

Epithelial-mesenchymal transition (EMT) is a reversible plethora of molecular events where epithelial cells gain the phenotype of mesenchymal cells to invade the surrounding tissues. EMT is a physiological event during embryogenesis (type I) but also happens during fibrosis (type II) and cancer metastasis (type III). It is a multifaceted phenomenon governed by the activation of genes associated with cell migration, extracellular matrix degradation, DNA repair, and angiogenesis. The cancer cells employ EMT to acquire the ability to migrate, resist therapeutic agents and escape immunity. One of the key biomarkers of EMT is vimentin, a type III intermediate filament that is normally expressed in mesenchymal cells but is upregulated during cancer metastasis. This review highlights the pivotal role of vimentin in the key events during EMT and explains its role as a downstream as well as an upstream regulator in this highly complex process. This review also highlights the areas that require further research in exploring the role of vimentin in EMT. As a cytoskeletal protein, vimentin filaments support mechanical integrity of the migratory machinery, generation of directional force, focal adhesion modulation and extracellular attachment. As a viscoelastic scaffold, it gives stress-bearing ability and flexible support to the cell and its organelles. However, during EMT it modulates genes for EMT inducers such as Snail, Slug, Twist and ZEB1/2, as well as the key epigenetic factors. In addition, it suppresses cellular differentiation and upregulates their pluripotent potential by inducing genes associated with self-renewability, thus increasing the stemness of cancer stem cells, facilitating the tumour spread and making them more resistant to treatments. Several missense and frameshift mutations reported in vimentin in human cancers may also contribute towards the metastatic spread. Therefore, we propose that vimentin should be a therapeutic target using molecular technologies that will curb cancer growth and spread with reduced mortality and morbidity.

## 1. Introduction

Epithelial to mesenchymal transition (EMT) is a reversible biological process in which epithelial cells lose their unique features of apicobasal polarity, epithelial markers, intercellular junctions, reorganization of the cytoskeletal architecture, immobility and differentiation and redirect to mesenchymal phenotype with the ability to migrate and invade [1]. EMT can be of three different types based on pathophysiological tissue context: type-1 EMT is an important physiological event during organogenesis and embryonic development, such as gastrulation or the outmigration of various cell types from the neural crest; type-2 EMT happens during wound healing for the induction of cell migration, growth and organ fibrosis [2] and type-3 EMT is described in the initiation and progression of multiple pathologies, including cancer and metastasis. In different carcinomas, EMT is characterized by the migration of epithelial cancer cells to invade the distant body sites by transforming into cells with the mesenchymal phenotype [3]. Cells previously activated by the EMT programme often revert to the epithelial state; this mechanism is called mesenchymal–epithelial transition (MET) [4]. In addition to the classical concept of EMT/MET in cancer cells, a recent concept of partial EMT (EM) was introduced, in which cells simultaneously express both epithelial and mesenchymal hybrid features [5,6]. This hybrid state makes them metastable, which is a dynamic state enabling cancer cells to induce or revert to EMT. Cancer cells may stably acquire one or more hybrid EM phenotypes expressing mixture of epithelial and mesenchymal traits. This multishaded EMT concept is known as epithelial–mesenchymal plasticity [7]. Researchers have categorized the hybrid EM into early and late types. The cells in early hybrid EM express both epithelial (cytokeratins) and mesenchymal (vimentin) markers but are less adhesive and rounded in shape. In the late hybrid stage, the mesenchymal markers become more pronounced and the epithelial phenotype is suppressed. Their shape becomes elongated, and adhesion is completely lost. Late hybrid EM stage can lead into a stable mesenchymal state [8].

**Figure 1:**
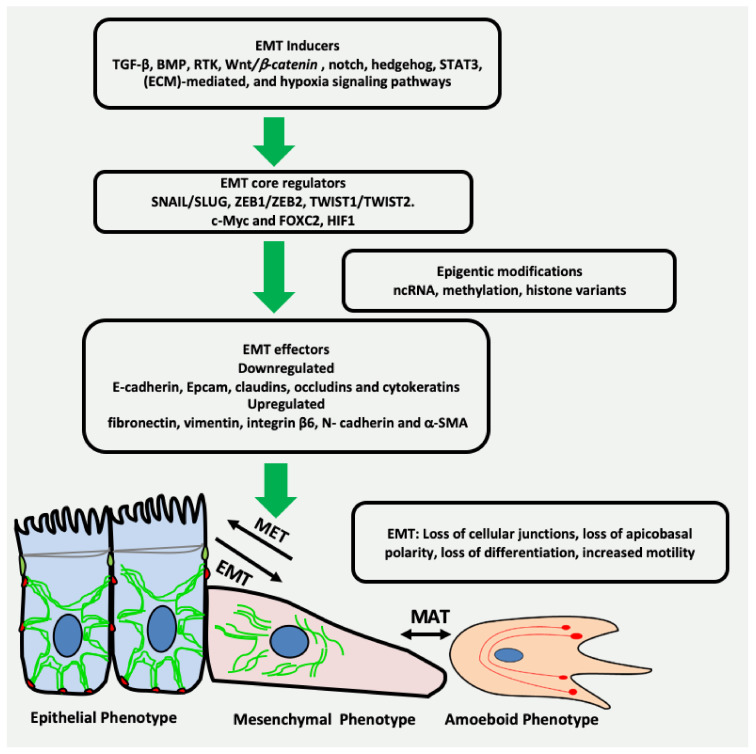
Key events in EMT. Multiple growth factors, microenvironmental factors and other EMT inducers activate the transcription factors related to EMT. As a result, there is downregulation of the genes related to cell junctions and differentiation. Moreover, the genes specific to mesenchymal phenotype such as vimentin are upregulated, resulting in loss of intercellular junctions, apicobasal polarity, differentiation and increased cell motility, ultimately leading to cancer invasion. ncRNA = non-coding RNA.

The phenomenon of EMT is an intricate process with a timely interplay of a variety of complex network comprising inducers, core regulators and effectors [9]. EMT inducers include transforming growth factor-beta (TGF-β), bone morphogenetic protein (BMP), receptor tyrosine kinase (RTK), Wnt/*β*-catenin, NOTCH, hedgehog, signal transducer and activator of transcription 3 (STAT3), extracellular matrix (ECM)-mediated, and hypoxia signalling pathways (Figure 1) [10,11,12]. These EMT inducers lead to expression and functional activation of EMT core regulators, which among others include three major groups of EMT-activating transcription factors (EMT-TFs): the Snail family of the zinc-finger transcription factors Snail/Slug, the zinc-finger E-box binding homeobox (ZEB) family of transcription factors ZEB1/ZEB2, and the Twist family of basic helix-loop-helix (bHLH) transcription factors TWIST1/TWIST2 [13]. Other EMT-TFs are c-Myc, FOXC2 and HIF1. The activation of EMT-TFs is further fine-tuned by epigenetic modification leading to the induction of the expression of several EMT effectors that define the identity of the cell [14]. The epithelial biomarkers, such as E-cadherin, EpCAM, claudins, occludins and cytokeratins are downregulated, whereas the mesenchymal markers such as fibronectin, vimentin, integrin β6, N-cadherin and α-SMA are upregulated [15]. The key events during EMT are summarized in Figure 1. 

Vimentin is an important type III intermediate filament (IF) protein alongside other cytoskeletal components, such as microfilaments and microtubules. Its dynamic role in different fundamental cellular processes such as structural support, attachment, migration and signalling is widely accepted [16]. Vimentin is consistently observed to be overexpressed during cancer metastasis and is therefore generally acknowledged as a canonical biomarker of type-3 EMT [17,18]. Several studies have highlighted its central role in the regulation of this complex process [19,20]. Vimentin filaments protect the cancer cells from mechanical stresses during the migration or squeezing through narrow spaces by providing a viscoelastic framework and support the positioning and integrity of organelles, especially the nucleus, during EMT and cancer progression [21]. In addition, it is reported that vimentin protects the cancer cells from the internal stress of misfolded proteins by directly binding to stress granules and aggresomes, supporting their subsequent destruction [22]. However, the exact mechanism used by vimentin to perform these functions is not known and requires further investigation.

In this review, we discuss various aspects of the pathophysiological mechanisms and regulation of type-3 EMT, and the driving role of vimentin as an upstream and/or downstream effector in signalling feedback loops in regulating and sustaining EMT, cancer invasion and metastasis.

## 2. EMT May Produce Cancer Stem Cells (CSCs) Expressing Vimentin

**Figure 2:**
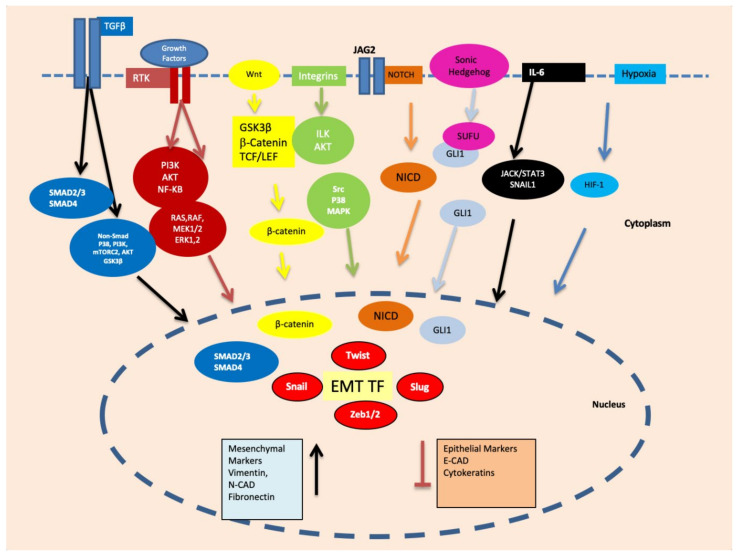
Important signalling pathways, such as Smad, PI3K/mTORC2/AKT/GSK3β, RAS/RAF/MEK1/2/ERK1, PI3K/AKT/NF-κB, ILK/AKT, Src/P38/MAPK, NOTCH, GLI1/SNAI2, JACK/STAT3 and HIF-1 are activated during EMT via growth factors, hypoxia and other microenvironmental factors. These signalling pathways ultimately upregulate the transcription factors related to EMT such as Snail, Slug, Twist, ZEB1 and ZEB2.

Cancer stem cells (CSC) are a small population of cells capable of self-renewal, which are known to resist therapeutic interventions and immune responses. Being pluripotent, these cells can provide cellular seeds to initiate new tumours at distant sites [23]. It was proposed that EMT can transform non-CSCs into cancer stem cells, which are invariably vimentin-positive [24]. In addition, it is believed that CSCs are generated as a result of adaptations and crosstalks with a tumour microenvironment, as well as in therapeutic interventions resulting in the generation of a heterogeneous subpopulation. Hypoxic conditions particularly contribute to the development of CSC characteristics including self-renewal, EMT, and drug resistance [25]. Hypoxia-inducible factors (HIFs) are the primary mediators of cellular responses, such as proliferation, EMT and metastasis, to hypoxic conditions [26]. Several other pathways implicated in the regulation of stemness phenotypes via HIFs include the TGF-β [27], Wnt/β-catenin [28], TNFα and NF-κB signalling [29]. These signalling cascades are also implicated in the induction of EMT via the transcriptional control of EMT-associated transcription factors, such as SNAI1, TWIST, ZEB1, SLUG and TCF3 leading to vimentin expression, as described above (Figure 2).

Cancer stem cells can exist in epithelial, mesenchymal or hybrid (mixed) states. They have the ability to switch between these different cellular states to maintain their survival, escape the immunity and grow at secondary sites. This switching is conducted by the complex interactions of various transcription factors, signalling pathways and microenvironmental factors [30]. The epithelial-like state of CSCs is characterized by the downregulation of mesenchymal markers, such as vimentin, and the upregulation of epithelial markers, such as CDH1. The expression of these markers is reversed in the mesenchymal state of CSCs. Both epithelial and mesenchymal markers are expressed in hybrid state [31]. CSCs express different cell surface proteins, such as CD34, CD44, CD24 and CD133, transcriptional factors, such as SOX2, NANOG, OCT ¾, SALL4 and other proteins that are not characterised as cell surface proteins, or transcription factors such as, ALDH, BMI1, Nestin and CXCR. These diverse markers are used to distinguish CSCs from the rest of the tumour population in different cancers [32]. These markers are expressed in a tissue-specific manner e.g., CD44, CD24 and ALDH are specific to breast cancer, CD34, CD8 to leukaemia, CD133 to colon cancer, CD44 to head neck cancer and CD90 to liver cancer [33]. These markers can be variably expressed according to the state of the CSCs. For example, in breast cancer stem cells, the CD24-CD44+ signature is related to a mesenchymal state with higher vimentin expression while the ALDH+ signature corresponds to an epithelial state of the cancer stem cells [31]. In normal cells, CD44 is a glycoprotein receptor for hyaluronic acid that is involved in cell adhesion, proliferation, differentiation, migration, angiogenesis and cell survival [34]. It is overexpressed in a variety of cancers, such as breast, colon, bladder, gastric, glioma, head and neck, prostate and leukaemia [32]. A soluble form of CD44 exists and is also overexpressed in certain cancers [35]. This soluble CD44 can bind to the vimentin head domain on the surface of endothelial cells, which is consistent with the fact that both CD44 and vimentin are overexpressed in oral squamous cell carcinoma (OSCC) and prostate cancer; however, the molecular basis of this association has not been fully elucidated [35]. CD24 is a cell adhesion sialoglycoprotein identified as a differentiation marker for hematopoietic and neuronal cells [36]. A higher CD44/CD24 ratio is positively correlated with vimentin in the breast CSC population [37]. ALDH1 (specifically isoform ALDH1A1) is another recently identified CSC marker in different tumours [38,39,40] and regulates the oxidation of retinal substrates into retinoic acid [40]. The increased expression of ALDH1 is related to the MET state of CSCS expressing lower levels of vimentin in breast cancer [41]. The sex-determining region Y-box 2 (SOX2) is an important transcription factor essential for the potential of stem cell multi-lineage. It can reprogram primary cells into stem cells [42], its levels are frequently upregulated in carcinomas such as HNSCC [43] with an inverse correlation between SOX2 and vimentin expression [44], and with a loss of SOX2 inducing tumour invasion through the upregulation of vimentin expression [45]. The possible role of miR-378 in the SOX2/Vim inverse functional relationship was also reported [31,46].

## 3. Vimentin Expression during Mesenchymal–Amoeboid Transition (MAT) of CSC

The concept of amoeboid movement was taken from amoeba *Dictyostelium discoideum* that rapidly move via contraction and expansion of the cell body without integrin interactions with the substrate. The belief that the amoeboid movement of cancer cells is linked to their invasive potential was first proposed in 1867 [47,48,49]. Different patterns of cell motility in cancer cells have been proposed by researchers and activated on an ad hoc basis during cancer invasion, which may also coexist within a population. This “switching of migration modes” is termed as “plasticity of cell motility”, which is considered imperative for cancer invasion [50]. One of the most familiar classifications of cell motility is individual versus combined cell migration that is further segregated based on mesenchymal or amoeboid phenotype. The single-cell motility-based “plasticity of migration” involves mesenchymal-to-amoeboid (MAT) and amoeboid-to-mesenchymal transitions (AMT) (Figure 1) [51]. A similar pattern could be observed in multicellular combined motility as the “plasticity of migration”, and involves collective-mesenchymal transition (CMT) and collective-amoeboid transition (CAT). As both CMT and CAT are reversible, therefore the terms, mesenchymal-collective transition (MCT) and amoeboid-collective transition (ACT), were also proposed [52]. During collective cell migration, a heterogeneous population of cells move together; the leader cells move by amoeboid movement while other members express mesenchymal phenotype and have intercellular connections [52].

Amoeboid cancer cells, unlike mesenchymal ones, migrate through the ECM barrier without proteolytic degradation of ECM and integrin clustering. As the amoeboid cells are rounded or ellipsoid, highly deformable, lacking focal adhesions and exhibit minimal cell-matrix contact, they move much faster than the mesenchymal cells [53,54]. Their nuclei are highly deformed, compressed and shifted towards the leading edge to allow movement through narrow spaces in ECM [55]. An amoeboid pattern of motility is reported when the cancer cells migrate through a soft medium such as blood or the lymphatic system [53]. Amoeboid movement is generally defined as a ‘path finding’ rather than the ‘path generating’ movement of mesenchymal cancer cells [52,56]. The detailed molecular basis of mobility shift from mesenchymal to amoeboid movement is not clear. Most studies are unable to describe the true molecular signature and transcriptional regulation of MAT in relation to tumour microenvironment and host immune response [51,57]. However, cellular stiffness, density, and other ECM factors along with the presence of chemotactic agents may be the determining factors in switching between mesenchymal and amoeboid states of cell motility [54]. A flexible vimentin network is reported to support the amoeboid mode of cell motility by conferring viscoelastic properties to the cell, protecting the nucleus and DNA from damage during propulsive squeezing movements [58]. Breast carcinoma cells devoid of vimentin are reported to be less contractile and less effective in migration [59]. In 3D cell cultures, vimentin is a prerequisite to the generation of propulsive pressure necessary to drive cell migration through confined spaces and vimentin knockdown leads to defective migration [59].

During metastasis, cancer cells transition from epithelial to mesenchymal phenotype or vice versa until they find suitable secondary host sites. Earlier reports suggested that stem cell-like features in cancer cells were induced by EMT; however, the latest research has linked the gain of stemness to cellular plasticity [24,60]. There are published reports proclaiming that EMT alone is not a prerequisite for cancer metastasis [61] and targeting EMT alone may lead to chemoresistance relapse, therefore both EMT and MET should be therapeutically targeted. Whether metastasis requires EMT or not is debatable; however, it is certain that EMT and metastasis both lead to the expression of vimentin [54,56,62].

## 4. Transcription Factors Inducing EMT

Induction of EMT requires the expression and activation of several transcription factors and associated proteins shown in Figure 2.

### 4.1. Snail and Slug

SNAI1 (Snail) and SNAI2 (Slug) are the two members of the Snail family of transcription factors which are activated by signalling cascades including TGF-β, RTKs, Wnt, NOTCH, BMPs, and TNF [63,64], in order to induce EMT by repressing E-cadherin, leading to the loss of cellular junctions and increased cell migration [65]. Furthermore, they are implicated in cellular differentiation and survival events due to their anti-apoptotic potential and control of the cell cycle via cyclin D/p21 [66]. Their post-translational modifications such as phosphorylation via the large tumour suppressor kinase 2 (Lats2) or glycogen synthase kinase 3β (GSK3β) were described as the controlling factors in their subcellular localization and protein expression. It was reported that vimentin regulates Snail and Slug expression using a feedback loop, and downregulation of vimentin consistently reduces their mRNAs as well as affects their protein expression [59,67]. It also serves as a downstream effector of the Snail or Slug-induced EMT to enhance cell migration [59]. A vimentin/extracellular signal-regulated kinase (ERK) axis was reported to provide a supporting framework to recruit Slug and enhance its phosphorylation at Ser-87, which is a prerequisite for EMT induction [67].

### 4.2. TWIST1

The Twist protein, encoded by the *TWIST1* gene, is a key transcription factor that regulates EMT and cancer metastasis by activating the genes linked to mesenchymal lineage, such as vimentin, in contrast to the Snail superfamily, which mainly represses the epithelial-specific genes, such as E-cadherin [68]. Its active role in EMT, angiogenesis, cancer stem cells population maintenance, cancer invasion, metastasis, chromosome instability and apoptosis inhibition is well documented [59,68]. Its expression can be induced via the signal transducer and activator of transcription 3 (STAT3), Akt/PKB, Ras, mitogen-activated protein kinase MAPK and Wnt signalling pathways [69]. Vimentin is the downstream effector of Twist in EMT-induced molecular cascades. The exact mechanism by which Twist upregulates vimentin expression is still not completely understood.

### 4.3. ZEB1 and ZEB2

The Zinc-finger E-box binding homeobox (ZEB) family is comprised of two members, ZEB1 and ZEB2/SIP1. They are the critical regulators of type-3 EMT and cancer invasion. Their role as a gene repressor or activator of EMT is dependent on their conformation, transcription levels and promoter of the target gene [70]. They bind to the E-Box sequence in the promoter of E-cadherin and downregulate its expression to support cell migration [71]. Vimentin is the downstream effector of ZEBs in multiple EMT-related signalling pathways. Moreover, ZEB1 can bind to specific sequences as the promoter of vimentin to control its transcription and mRNA levels [72]. The ZEB2/Smad interacting protein-1, SIP1, also indirectly regulates vimentin expression; however, the precise underlying mechanism is unknown [73].

### 4.4. c-MYC

c-Myc is a pivotal “master” transcriptional factor that regulates the expression of multiple genes involved in cell proliferation, differentiation, angiogenesis, apoptosis, metabolism, EMT, invasion and metastasis [74]. It can regulate vimentin expression and associated pathways for EMT induction and cell migration [75]. Oncogenes such as c-Myc can induce cellular stiffness and confer invasive potential to cancer cells via HDAC6-dependent deacetylation of α-tubulin and reorganization of the vimentin network. This reorganization involves a union of peripheral thin filaments into thicker fibers that later accumulate in the perinuclear region. The authors claim that increased stiffness of the cells due to reorganized vimentin can increase their invasive potential in adherent culture [76].

### 4.5. HIF-1

Hypoxia-inducible factor-1 (HIF-1) is responsible for hypoxia-related cellular responses, cancer microenvironmental modulation and EMT induction. It also regulates vimentin at the transcriptional levels and modulates cell migration during cancer invasion [77].

## 5. Regulation of Vimentin by Epigenetic Factors

### 5.1. DNA Methylation

Epigenetic factors are the emerging candidates that are shown to regulate EMT by regulating the EMT-TFs and EMT-effectors. Four common epigenetic molecular mechanisms are DNA methylation, chromatin remodelling or histone modifications, histone variants and noncoding RNA regulation [78]. Aberrant epigenetic enzyme activity in cancers results in abnormal DNA methylation and chromatin remodelling which then results in increased invasiveness and metastasis. The Pro-X-Asp-Leu-Ser (PXDLS) motif of both ZEB TFs, ZEB1 and ZEB2, is used to recruit epigenetic silencing complexes for regulation of the transcription of target genes [79]. Histone acetylation and deacetylation are the important epigenetic mechanisms for gene regulation. An important member of the HDAC family is histone deacetylase 6 (HDAC6), which targets both histone and nonhistone substrates, such as α-tubulin, cortactin and heat shock protein 90 [80]. It enhances the cell motility in cancer metastasis through catalyzing α-tubulin deacetylation [81]. Rathje and colleagues have shown that under the influence of oncogenes, HDAC6 reduces the acetylation of the microtubules at the cell periphery, which leads to a reorganization of the vimentin IF network and a consequent increase in stiffness at the cell periphery. This increased stiffness was associated with increased cell invasiveness in their experimental model that is contrary to the reports showing that cellular deformability increases the cell invasiveness. They have further compared that data published for linking cell invasiveness with increased deformability is for non-adherent cells and may be different for adherent cells [76]. The DNA methylation of the epithelial gene promoters creates highly stable methylated CpG dinucleotides that can be propagated to subsequent cell generations [82]. Vimentin expression in EMT is highly influenced by the methylation of its promoter region with an inverse effect on its expression and disease progression in gastric cancer [59].

### 5.2. MicroRNAs and Non-Coding RNAs

MicroRNAs (miRNAs) are single-stranded, endogenous, non-coding regulatory RNAs, approximately 18–22 nucleotides long, with the ability to regulate many biological processes, such as cell proliferation, cell cycle regulation and apoptosis in normal tissues by binding to the 3′-untranslated region (3′-UTR) and degrading the mRNA of target genes. MicroRNAs play a major role in EMT by regulating the main EMT transcription factors, ZEB1, ZEB2, Snail, Slug, TWIST1, and FOXC2 as well as the EMT effectors themselves. Moreover, a single miRNA can target mRNAs of multiple genes, either from the same pathway or across diverse pathways, leading to global changes in the expression patterns. In addition, a large number of miRNAs appear to act in a synergistic manner, leading to significant amplification of protein expression for ZEBs/Snails/Twist and EMT effectors to influence the course of EMT [83].

The expression of vimentin is regulated by non-coding RNA (ncRNA) either directly or indirectly and is subject to complex regulatory feedback signals. A list of miRNAs and ncRNA that bind directly to the 3′ UTR of vimentin and regulate its expression are listed in Table 1. Regulation of these ncRNAs was shown to affect the expression of vimentin, thereby influencing the EMT characteristics. For example, the testicular nuclear receptor (TR4) downregulates miR-490-3p by binding directly to TR4-response-elements (TR4REs) on miR-490-3p, which increases vimentin mRNA [84]. During hypoxia, the hypoxia-induced factor-1α (HIF-1α) induces the downregulation of miR-548an in pancreatic cancer cells to induce vimentin expression. HIF1α forms a complex with histone deacetylase 1 (HDAC1) and binds to the hypoxia response elements (HRE) on the miR-548an promoter resulting in its downregulation, which subsequently increases vimentin expression [85].

## 6. Role of Vimentin in Anoikis and Anastasis

**Figure 3:**
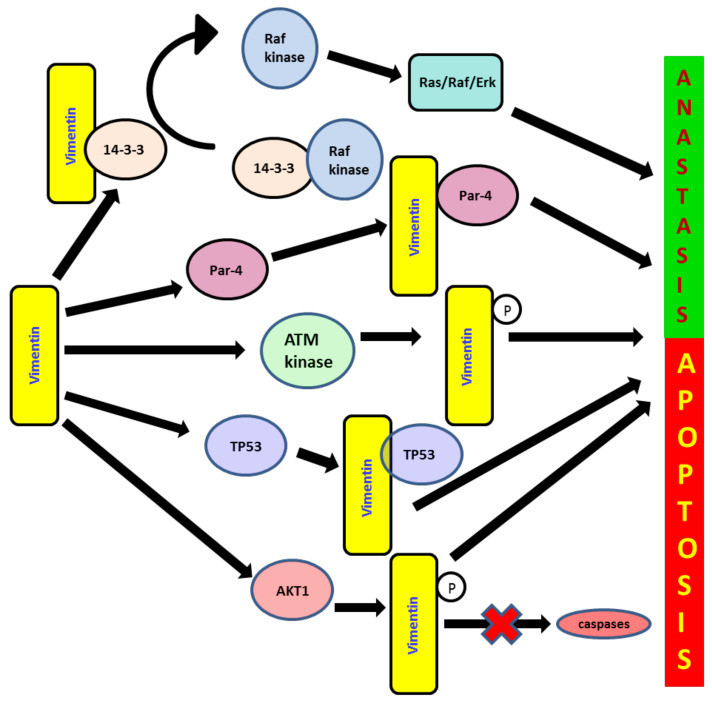
Loss of apoptosis and anastasis, “recovery from the brim of death”, are the important tools employed by cancer cells to progress, resist therapeutic agents and sustain the complex microenvironmental factors. Vimentin deregulates apoptosis and consequently supports cancer growth via its interaction through 14-3-3 protein-mediated mechanisms and prevents its free availability to regulate different molecular cascades of apoptosis, cell cycle and cancer development via oncogenic Raf, Bad, Bax Cdc25 and AKT interactions. It also binds to the protein prostate apoptosis response-4 (Par-4), preventing its secretion and consequently disrupting its apoptotic activities. It is a key downstream effector of ATM-mediated signalling cascades to inhibit apoptotic events during the DNA damage response to chemotherapeutic agents, and it upregulates early cell migration and the related escape from apoptotic death. It also induces nuclear translocation of cytoplasmic p53, thus causing downregulation of p53-mediated apoptosis.

Anoikis is the invocation of apoptosis and is induced upon cell detachment from the extracellular matrix. Anoikis resistance is one of the tools employed by cancer cells for their survival. It is one of the key pathways activated for cancer cell death by different therapeutic agents [104] (Figure 3). During apoptosis, major cytoskeletal deformation is mediated by the cleavage of vimentin by caspases (especially caspases 3, 6, 7) leading to cell death [105]. Apoptosis induced by various stimuli leads to vimentin being preferentially cleaved by multiple caspases at distinct sites in vitro, including Asp^85^ by caspases-3 and -7 and Asp^259^ by caspase-6, yielding multiple proteolytic fragments. This disrupts vimentin as well as other cytoplasmic IF networks. During the caspase 3/7 mediated proteolysis of vimentin, the N- terminal 1-85 fragment with pro-apoptotic activity is generated, which amplifies the pro-apoptotic signal to promote apoptosis [105].

Recently, the term anastasis was coined to define a process, wherein cancer cells undergoing programmed cell death develop resistance and revert to a normal physiological state once pro-apoptotic agents, such as chemotherapeutic drugs, are removed from the system (Figure 3) [106]. It encompasses both pro-survival and pro-metastatic activities. It is widely accepted that EMT is a possible escape mechanism employed by cancer cells, in order to avoid late phases of apoptosis and resist therapeutic agents [107]. A pro-tumorigenic role of vimentin was observed through its effect via the PI3K/AKT signalling pathway. AKT1 activation induces soft-tissue sarcoma (STS) cell motility and invasiveness partially due to vimentin phosphorylation at S39 [108]. Vimentin is also reported to block the sequestration of Raf kinase through its interaction with the 14-3-3 protein, thus maintaining the pro-proliferative Ras/Raf/Erk signalling pathway [109]. It interacts with the 14-3-3 protein, which prevents its free availability to actively participate in different molecular cascades of apoptosis, cell cycle regulation and cancer development via oncogenic Raf, Bad, Bax Cdc25 and AKT interactions [110]. It maintains phosphorylated ERK levels by inhibiting phosphatases, thus keeping various ERK mediating cell signalling active, which consequently supports EMT and cancer progression [67]. AKT1 also directly phosphorylates vimentin and protects its degradation; intact and free vimentin is then available to enhance cell migration and invasion. Vimentin also binds to the prostate apoptosis response-4 (Par-4) protein, preventing its secretion and thereby disrupting its apoptotic activities [111]. Vimentin also interacts with cytoplasmic p53 to translocate into the nucleus, thus causing the downregulation of p53-mediated apoptosis [112].

## 7. Role of Vimentin in the DNA Repair System during EMT

The cancer genome accumulates mutations due to defective DNA repair mechanisms. In normal cells, the DNA repair machinery puts a temporary pause on the cell cycle, allowing the cells to repair the damaged DNA and escape apoptosis. The genes associated with the DNA repair mechanism are also associated with the gain of migratory properties and EMT [113]. During EMT and metastasis, the hyperactive DNA damage repair system can enable the cancer cells to resist environmental stresses [114].

Vimentin is reported to be a nuclear matrix protein and is actively involved in all the nuclear matrix-associated mechanisms, such as DNA repair, transcription and replication. It can organize chromatin and preserve genomic as well as mitochondrial DNA [115]. It interacts with many proteins of the DNA repair system. It was reported that when EMT is induced by the DNA damaging agent camptothecin (CPT) in colorectal and lung carcinoma cell lines, the ATM kinase, “a DNA damage sensor” is upregulated, which subsequently stimulates Snail. Vimentin, as a key downstream effector of ATM, mediates the signalling cascades to cease the apoptotic events during the DNA damage response to CPT and upregulates early cell migration, resulting in the cells escaping apoptotic death and gaining migratory potential. This suggests that vimentin via AKT may be a key molecule to resist apoptotic death and induces cell migration during EMT [116].

Poly (ADP-ribose) polymerase 1 (PARP-1) is a critical multifunctional nuclear enzyme that regulates DNA repair, apoptosis and chromatin structural organization. Its upregulation is reported in tumour cells to induce EMT via active DNA repair. It can directly bind to the vimentin promoter and upregulate its expression thereby playing an indirect role in inducing EMT [117]. The DNA-dependent protein kinase, DNA-PK, when activated by stable DNA double-strand break-mimicking molecules (Dbait32Hc), can also target vimentin by phosphorylating S459. However, the phosphorylated vimentin located at cell protrusions can reduce the cell migratory and adhesive potential [118]. In ovarian cancer the downregulation of vimentin upregulates the 14-3-3 protein/ Cdc25C-mediated interactions that consequently inactivate Cdk1, leading to persistent G2 cell cycle arrest. This prolonged arrest enables the cells to repair DNA damage induced by drugs such as cisplatin. These observations indicate that vimentin may be indirectly involved in the regulation of the cell cycle [119].

## 8. Vimentin Regulating Other Genes during EMT

**Figure 4:**
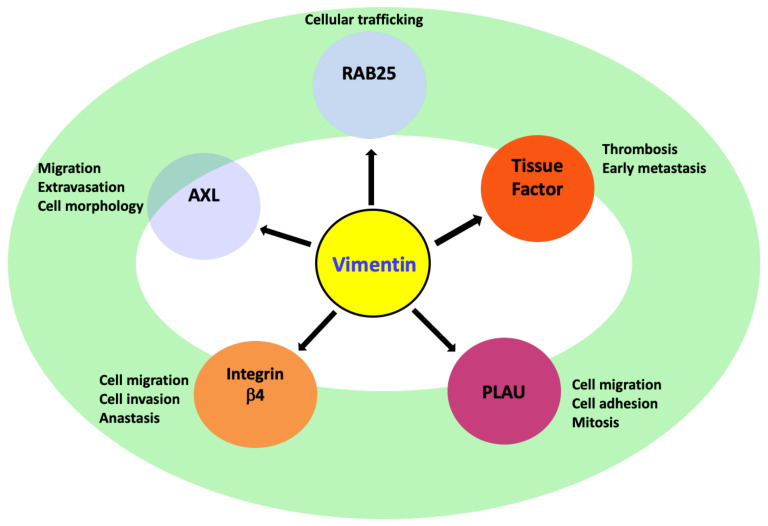
Vimentin supports cell migration and early cancer invasion by transcriptional regulation of different genes and activating multiple signalling pathways, such as RAB25-mediated AKT/GSK-3β/Snail-signalling, pro-migratory enzyme AXL-mediated ERK signalling, integrin β4-mediated PI3K/AKT/MAPK-signalling and PLAU /ERK1/2/PI3K-signalling or PLAU-mediated TGF-β1/MMPs genes transcriptional upregulation.

The vimentin promoter contains an NF-κB binding motif [120], a TGF-β1 response element [121], binding sites for the key regulators of EMT from the Smad family [122] and an AP-1/Jun binding motif [123]. Vimentin expression is also transactivated by β-catenin/TCF binding to the *VIM* promoter [124]. Vimentin directly regulates the expression of many genes associated with EMT to induce cell migration and metastasis (Figure 4). However, there are limited studies (clinical and non-clinical) showing the direct impact of vimentin on EMT associated genes [19,20] and therefore further research is required in this area.

### 8.1. AXL

Receptor tyrosine kinase *AXL* gene is a member of the tumour-associated macrophages (TAM) family and encodes Axl kinase. The supporting role of Axl kinase in cell migration designates it as a “pro-migratory-enzyme” [125]. Moreover, it also helps the cancer cells to escape the immune response, thus making it a crucial enzyme linked to EMT and metastasis [20]. It is also an upstream regulator of important signalling pathways active during EMT, such as JAK/STAT, PI3K-AKT-mTOR, NF-κB and MEK/ERK [126]. The vimentin-ERK axis is critically reported to regulate *AXL* expression [20]. Induction of EMT in the breast cancer cell line, MCF10A, by Slug and the oncogenic H-Ras, induces vimentin overexpression, which in turn leads to *AXL* expression and activation. A similar positive correlation between vimentin and AXL expression was observed in breast cancer patients as well as in 92 breast cancer cell lines. Therefore, the crosstalk between vimentin and AXL plays a key role in vimentin-dependent cancer cell migrations observed during EMT [20,126].

### 8.2. Integrin β4 (ITGβ4)/CD104

*Integrin β4 (ITGβ4)*/CD104 gene encodes the integrin β4-subunit, which is expressed in squamous epithelial cells, Schwann cells, fibroblasts, endothelial cells etc. [127]. It only binds with the α6 subunit to form a heterodimeric integrin known as α6β4: a cell adhesion molecule that functions as an extracellular matrix ECM receptor for laminin-5 (basal lamina protein) and supports the hemidesmosomes formation in epithelial cells [63,127]. During EMT and cancer progression, when hemidesmosomes are lost, the cytoplasmic domain of ITGβ4 is phosphorylated, which releases the integrin α6β4 from the hemidesmosomes and freely interacts with different growth factors receptors such as the epidermal growth factor receptor (EGFR). This interaction further stimulates downstream signalling pathways, such as PI3K, AKT and MAPK involved in EMT, cancer progression and metastasis [128]. Alongside this, there is also the RhoA and small GTPases activation that regulates the actin cytoskeleton involved in cell migration, formation of filopodia and invadopodia [127]. Furthermore, ITGβ4 also interacts with other EMT related genes such as Autotaxin (ATX), also known as ectonucleotide pyrophosphatase/phosphodiesterase 2 (NPP2 or ENPP2), S100A4 and transcriptional factors, such as nuclear factor kappa B (NF-κB) and nuclear factor of activated T-cells (NFAT) [63]. Vimentin silencing leads to the suppression of *ITGβ4* mRNA and its protein, which suppresses cell migration and the invasive potential of the cancer cells [19,20]. Therefore, during complex EMT events, increased vimentin expression (and its phosphorylated version) may regulate cell migration, cancer invasion and apoptosis evasion through the *ITGβ4* transcriptional regulation and associated multiple signalling pathways, highlighted above [63].

### 8.3. PLAU

Plasminogen activator urokinase (*PLAU)* encodes urokinase serine protease, also called a urokinase-type plasminogen activator (uPA). uPA converts plasminogen to its active form, plasmin, which further supports tissue degradation and angiogenesis [129]. Urokinase also plays an active role in cell migration, adhesion and mitosis during EMT and cancer progression through associated signalling cascades, specifically ERK1/2 and PI3K pathways, or directly, through increased transcription of TGFβ1 and matrix metalloproteinases (MMPs) genes [130]. In addition, it interacts with other transmembrane protein receptors, such as vitronectin, integrins, G-protein-coupled chemotaxis receptors and tyrosine kinase receptors, especially EGFR, in order to regulate cell migration [131]. ERK is reported to equally regulate uPA expression through the AP-1 transcription factor [130]. Vimentin is reported to regulate the transcription of *PLAU* and hence affects the levels of uPA protein. Silencing vimentin in EMT models consistently suppressed *PLAU* expression, implying that vimentin may be regulating EMT and cell migration via the modulating *PLAU* transcription and associated molecular cascades [20].

### 8.4. Rab-25

The Ras-related protein Rab-25 is an epithelial-specific small GTPase encoded by the gene *RAB25*. It is active in cellular trafficking and frequently downregulated in different cancers such as HNSCC, ovarian, breast, lung and renal [132]. Its role in cancer progression and metastasis is not fully understood. While some studies have reported it as a tumour suppressor, whereas others have highlighted its oncogenic role via activation of the AKT/GSK-3β/Snail signalling pathway and its expression is associated with a poor prognosis. Silencing vimentin in breast epithelial cells can upregulate *RAB25* [19]. The transcription factor ZEB2 activates the vimentin promoter through the Sp1 transcription factor during EMT induction and also directly binds to E-box sequences in the *RAB25* promoter to downregulate it [133]. Hence vimentin may modulate the *RAB25* expression either directly or indirectly during EMT induction; however, the precise molecular mechanism is still unclear and further investigation is required to decipher it [130].

### 8.5. Tissue Factor (TF)

CSCs have recently been associated with venous thrombosis in cancer patients [134]. Tissue factor (TF) is a 47 kDa membrane-associated glycoprotein, which has recently been identified as a link between the hemostatic system and cancer progression [135]. It is observed that vimentin stabilises TF mRNA, thus promoting coagulant activity and early metastasis [136].

## 9. Vimentin Protein Interactions and Cytoskeletal Reorganization Related to EMT

**Figure 5:**
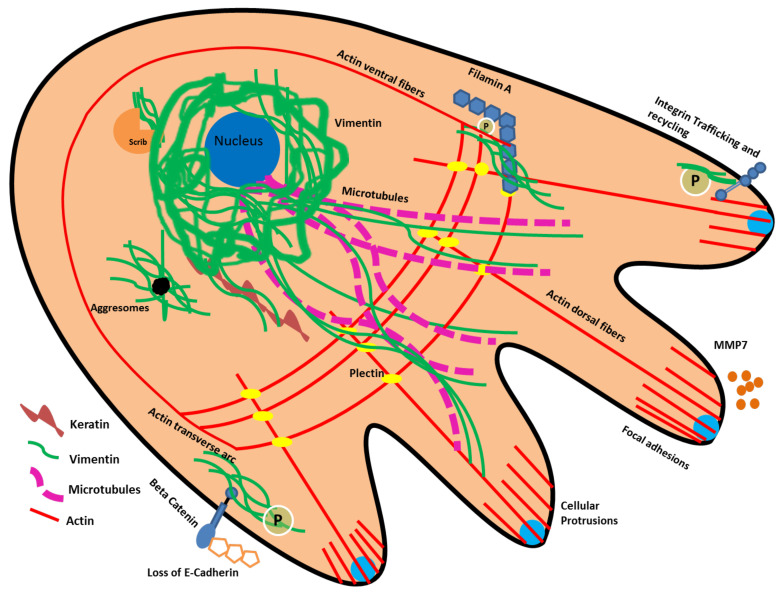
Vimentin works together with other cellular proteins to provide dynamic viscoelastic support to the cell and also actively behaves as a mechanosensor during cell migration in EMT. In migrating cells, it supports the functional role of cytoskeletal proteins, such as actin, microtubules and keratins, formation of new focal adhesions via integrins, ECM degradation via MT1-MMP, and the loss of intercellular junctions via EMT transcriptional regulators, such as Snail/Slug/Twist/Zeb1/2. In addition, it protects Scrib from proteasomal degradation and maintains its protein levels, thus supporting the cell migration through Scrib. Furthermore, it helps the cancer cells to survive internal stress by directly binding to stress granules and aggresomes, therefore supporting their subsequent destruction.

During EMT, the cancer cells acquire an elongated shape that typify the mesenchymal phenotype. This transformation in shape is primarily due to the loss of cellular junctions and reorganization of the cytoskeleton along the direction of migration. Vimentin, as an important member of the cytoskeletal protein family, interact with other cytoskeletal proteins to regulate cell migration, adhesion and spatial reorganization, and thereby behaves as a master regulator of EMT (Figure 5). It regulates cell migration by modulating the actin stress fibres via GEF-H1 and RhoA [137]. In an in vitro model of lung cancer, the activity of Rac1 (Ras-related C3 botulinum toxin substrate) was regulated by vimentin via VAV2 (a member of the guanine nucleotide exchange factor-VAV subfamily) to influence cellular junctions. Vimentin phosphorylation at S56 results in the disassembly of filaments into squiggles that are pivotal for lamellipodia formation through actin modulation via the Cdc42/Rac/p21-activated kinase (PAK) pathway. They are found at the leading edge of mature invadopodia [138]. Vimentin also strengthens actin attachment to new and pre-existing cell-matrix adhesions by interacting with filamin A, which is an actin crosslinking protein. In addition, it is reported that a direct interaction of the PKC-induced phosphorylated vimentin and filamin A in HEK-293 and 3T3 cells play a critical role in cell-spreading and beta 1 integrin upregulation; subsequently supporting cell migration [139]. Vimentin is directly attached to actin via its tail domain or indirectly via plectin, carmil and girdin [35] to regulate the mechanical integrity of the cell during cell migration [140,141].

Vimentin also interacts and reorganizes the keratin filaments and supports the desmosomal internalization and integrin recycling in cancers [59]. In the migrating cancer cells, the YRKLLEGEE motif in the rod sub-domain 2B of vimentin interacts with K14 to support cell migration [142,143]. In cancer cells undergoing EMT, higher vimentin levels are also linked with a dedifferentiated phenotype, whereas keratins are known markers of epithelial differentiation. How vimentin suppresses this keratin-based differentiation programme in epithelial cancers needs further investigation. One possible mechanism is the modulation of the K5/K14 pair expression through ΔNp63 in stratified squamous carcinomas such as OSCCs [144].

Vimentin also works together with microtubules (MT) to direct their accumulation near the nucleus; this particular interaction determines the direction of migration and maintains the polarity of the cell. When vimentin is knocked down, microtubules do not specifically localize in the perinuclear region; rather, their distribution is random and the cells lack directional migration [18]. However, the precise mechanism is not very clear. During active migration in wounded retinal-pigmented cells, vimentin is reported to serve as a template for “short-lived” microtubules and help the newly formed microtubules to arrange themselves along vimentin [145]. Vimentin IFs are long-lived and assemble themselves in such a way that the exact pattern of the earlier polarized microtubular network is copied. Therefore, when new microtubules are formed, vimentin guide their arrangement. Consequently, cell polarity is maintained during migration. The precise molecular interactions between vimentin and microtubules are not known. However, these interactions are either mediated through linker proteins such as adenomatous polyposis coli (APC) [146] or carried out via post-translational modifications e.g., phosphorylation [147]. Recently, it was observed that in endothelial cells vimentin is linked to microtubules via the Rudhira/Breast Carcinoma Amplified Sequence 3 (BCAS3) that stabilizes MT and helps in cell motility, angiogenesis and metastasis [35].

The hallmark of EMT is the loss of cell junctions mediated by the downregulation of E-cadherin, which allows the cancer cells to freely migrate. E-cadherin is an important transmembrane glycoprotein that maintains the cell-to-cell adhesion in epithelial cells [148]. The cytoplasmic domain of E-cadherin binds to actin filaments via α, β, and γ-catenins. Normally β catenin is preferentially located in E-cadherin complexes in order to support cell junctions [149]. However, during EMT and cancer progression, it accumulates in the cytoplasm and translocates into the nucleus, where it performs a transcription activator role in association with the family of T-cell factors (TCF)/lymphoid enhancer factor 1 (LEF1) in order to activate multiple genes involved in cell migration and cancer progression [124]. The vimentin promoter is mentioned as a possible target for the β-catenin/TCF pathway in order to enhance cell motility during EMT by increasing the vimentin expression in breast cancer cells [148,150].

Vimentin can be glycosylated and/or phosphorylated at multiple sites on its different domains; these modifications are pivotal for filament crosslinking and cell migration during EMT [145]. The phosphorylation of vimentin via PKCε upregulates integrin β1 trafficking and recycling; this crucial pathway is activated during EMT to enhance cell migration [151]. This is mediated by the interaction of the integrin β3 tail with a high level of vimentin accumulated underneath the plasma membrane. These interactions can lead to β3 clustering, resulting in enhanced integrin-mediated cell adhesion [151,152].

An important interaction of vimentin is with Scrib (a protein necessary for cell migration), which protects it from proteasomal degradation and maintains its protein levels, therefore increasing cell migration, maintaining cell polarity and enhancing cell proliferation [153]. Vimentin supports cancer cell survival at metastatic sites by supporting angiogenesis via NOTCH signalling [124] and calpains (proteolytic enzymes) which cleave the amino-terminal of vimentin; leading to a pool of soluble vimentin. As a result, membrane type 1-matrix metalloproteinase (MT1-MMP) translocation to the cell membrane is facilitated and guides the angiogenic sprouting [154]. Preliminary reports have shown that vimentin can act as a ligand and NKp46 on natural killer cells may be a potential possible receptor. The possibility of vimentin secretion from cells was proposed; however, the mechanism is not clear [35]. The possible role of extracellular vimentin in secreted form and as a ligand in EMT and metastasis is an emerging and exciting area of research.

Although most vimentin is located in the cytoplasm, a detectable amount of vimentin is present in the nucleus, where it interacts with nuclear proteins and DNA [115,155,156,157]. It is believed that the nuclear vimentin does not form long filaments but instead exists as ULF or small filaments [157,158]. Vimentin can bind to G-quadruplex (G4) structures in the nucleus, formed at guanine-rich genomic sites, and thought to regulate the expression of genes involved in cell migration by changing the genome topology [157]. The deep sequencing of the different types of cancer genome has shown large number of mutations in vimentin (Table 2). The majority of these mutations are missense, with a few gene fusions and frame shifts spread throughout the vimentin polypeptide chain including the head, rod and tail domains. The effects of these mutations on EMT and cancer progression are not known. However, given the fact that vimentin is intricately associated with so many cytoplasmic and nuclear proteins, it is very likely that these somatic mutations would have a pronounced effect on cancer progression and metastasis. This aspect of EMT and cancer biology needs to be further investigated.

## 10. Effect of Microbial Pathogens and Chronic Inflammation on EMT

Microbial pathogens including viruses, bacteria and the associated chronic inflammation are also reported to induce EMT and upregulate related signalling pathways and transcriptional factors, especially the transforming growth factor β [150]/Smad, Akt/β-catenin and Ras-MAPK pathway [160] affecting cell migration, differentiation and proliferation. The role of vimentin in this process has not been very well studied [35]. Some studies have reported the facilitating role of vimentin in viral infections (such as hepatitis C and poliovirus) and associated chronic immune-inflammatory response [141], EMT and cancer [161]. The newly discovered coronavirus, SARS-CoV-2, can induce EMT-like changes via upregulation of ZEB1 and AXL in lung cancer cells [162]; vimentin is reported to be the prerequisite for the viral entry into the cell by acting as a co-receptor [163]. EMT induction in chronically inflamed tissue may be a menacing attempt by the cells towards healing the damaged tissue. The induction of EMT is now considered a merging point between chronic inflammation and cancer progression [164]. Vimentin can regulate lymphocyte inflammatory responses and apoptosis in a cancer microenvironment by interacting with other inflammatory proteins, including heat shock protein 90 (HSP90), transmembrane protein 4 (TMP4) and 14-3-3 protein epsilon (YWHAE). It is reported to be a ligand for Dectin-1, the non-toll pattern recognition receptor (PRR), on monocytes; the resultant activated monocytes can contribute to chronic inflammation. Vimentin may also be involved in regulating the inflammatory response of lymphocytes that are exposed to endotoxins produced by microbial pathogens [165].

## 11. Conclusions and Future Perspective

This review highlights the importance of vimentin as an active player in the EMT that is the hallmark of cancer metastasis/invasion and indicates the areas of future research. During EMT, vimentin reorganizes, mediates the signalling pathways, supports other cellular organelles due to its viscoelastic properties and directs cell migration by forming cellular protrusions, decreasing the cell adhesions and increasing the migratory capacity of cells. In addition, vimentin modifies DNA repair pathways to support EMT and enable the cells to withstand different kinds of stresses during the cancer invasion phenomenon. In addition, it is reported to be a hindrance in the differentiation process by activating self-renewable and pluripotency-related genes that also aid in cancer progression. A large number of mutations in vimentin have been identified in human cancers; however, their role in EMT or in cancer progression is still to be discovered. Vimentin appears to be at the centre of the EMT/MET pathway controlling the plasticity of the cancer cells; however, the molecular interactions regulating these mechanisms are largely unknown. The knowledge we have about the role of vimentin in cancer progression and metastasis is just scratching the surface; therefore, there is an urgent need to explore the functions of this protein in type-3 EMT as well as in the development of the CSCs of different tissues. Going forward, we need to develop new strategies for targeting this key protein for cancer treatment, suppression of EMT, cancer progression, invasion and metastasis. This will be of huge benefit to patients by reducing mortality and morbidity.

## Figures and Tables

**Table 1:** List of microRNAs and non-coding RNAs which are shown to downregulate vimentin by degrading its mRNA in different human cancer cell lines.

MicroRNA	Cell Lines	Cancer Type	References
miR-146a	ESCC CE81T cells	Esophageal squamous cell carcinoma	[86]
miR-515-3p	KYSE150-Luc-LM3, KYSE410-Luc-I6, KYSE150-Luc,43 and EC109 and NE1-E6E7	Esophageal squamous cell carcinoma	[87]
miR-17-5p	No Access/withdrawn	Colorectal cancer	[88]
miR-124-3p/ miR-138-5p	SK-Hep1 and Hep3B	Hepatocellular carcinoma	[89]
miR-1275	MGC803 (CRL-1739) and SGC-7901 (CRL-5822)	Gastric cancer	[90]
miR-30a-5p	HepG2, Huh7 and HEK-293T, HCT116	Hepatocellular carcinoma	[91]
miR-876-5p	CAL27 and HEK293T (293T), HNSCC WSU-HN4 and WSU-HN6	Head and Neck squamous cell carcinoma	[92]
miR-490-3p	Caki-1, Sw839, 786-0, A498, OSRC-2, ACHN, 769-P	Clear cell renal cell carcinoma	[84]
miR-215	SW480, HCT116,	Colorectal cancer	[93]
miR-1246,	PLC-PRF-5 and SMMC-7721, HEK-293T	Hepatocellular carcinoma	[94]
miR-578, miR490-5p	PLC-PRF-5 and SMMC-7721, HEK-293T	Hepatocellular carcinoma	[94]
miR-509-5p	Panc1 and KMP3, KP4-4, BxPC3, CFPAC1 and SU.86.86	Pancreatic cancer	[95]
miR-138-5p	AsPC-1, BxPc-3, Capan-1, Capan-2, CFPAC-1, PANC-1, MIA PaCa-2 and SW1990	Pancreatic cancer	[96]
miR-30c	Review article	Global	[97]
miR-320a	BGC-823	Gastric cancer	[98]
miR-17-3p	HepG2	Hepatocellular carcinoma	[99]
miR-17-5p	SW480, HT29, LoVo	Colorectal carcinoma	[88]
miR-30a	Hs578T and MDA-MB-231	Breast cancer	[100]
miR-378	U87	Global	[46]
miR-294	SGC-7901 and HGC-27,MFC	Gastric cancer	[101]
AOC4P	J7 and SK-Hep1	Hepatocellular carcinoma	[102]
lncRNA-Dreh	Hepa1-6	Hepatocellular carcinoma	[103]

**Table 2:** Mutations detected in vimentin in different human cancers. Data extracted from The Cancer Genome Atlas database (TCGA) [159].

Cancer Type/Study of Origin	Mutation	Mutation Type	Effect of Mutation on Copy Number	TCGA Sample ID
Oligodendroglioma	NPM1-VIM	GENE FUSION	Shallow Deletion	TCGA-FG-6692-01
Dedifferentiated Sarcoma	COL3A1-VIM	GENE FUSION	Diploid	TCGA-DX-A48N-01
Dedifferentiated Sarcoma	COL1A1-VIM	GENE FUSION	Diploid	TCGA-DX-A7EI-01
Myxofibrosarcoma	TNXB-VIM	GENE FUSION	Gain	TCGA-DX-AB2T-01
Cutaneous Melanoma	S100A6-VIM	GENE FUSION	Not known	TCGA-GN-A26C-01
Cutaneous Melanoma	HSPG2-VIM	GENE FUSION	Not known	TCGA-GN-A26C-01
Cutaneous Melanoma	E156K	Missense_Mutation	Not known	TCGA-BF-A5EQ-01
Cutaneous Melanoma	E156K	Missense_Mutation	Gain	TCGA-D3-A3MR-06
Cervical Squamous Cell Carcinoma	E153D	Missense_Mutation	Diploid	TCGA-IR-A3LK-01
Adrenocortical Carcinoma	A247P	Missense_Mutation	Gain	TCGA-OR-A5KB-01
Astrocytoma	M372V	Missense_Mutation	Diploid	TCGA-DU-6392-01
Astrocytoma	E407V	Missense_Mutation	Diploid	TCGA-DU-6392-01
Serous Ovarian Cancer	E407K	Missense_Mutation	Gain	TCGA-59-2348-01
Serous Ovarian Cancer	R320Q	Missense_Mutation	Not known	TCGA-23-2649-01
Serous Ovarian Cancer	R364L	Missense_Mutation	ShallowDel	TCGA-24-1849-01
Serous Ovarian Cancer	N283D	Missense_Mutation	Diploid	TCGA-25-2397-01
Glioblastoma Multiforme	A301T	Missense_Mutation	Diploid	TCGA-06-5416-01
Glioblastoma Multiforme	E95Q	Missense_Mutation	Shallow Deletion	TCGA-14-0866-01
Sarcoma **	V171F	Missense_Mutation	Diploid	TCGA-X9-A973-01
Lung Squamous Cell Carcinoma	F15L	Missense_Mutation	Diploid	TCGA-21-5782-01
Lung Squamous Cell Carcinoma	R222L	Missense_Mutation	Diploid	TCGA-33-4587-01
Lung Squamous Cell Carcinoma	E244K	Missense_Mutation	Diploid	TCGA-37-3783-01
Lung Squamous Cell Carcinoma	A332S	Missense_Mutation	Shallow Deletion	TCGA-39-5021-01
Lung Squamous Cell Carcinoma	E187G	Missense_Mutation	Shallow Deletion	TCGA-39-5036-01
Lung Squamous Cell Carcinoma	L340M	Missense_Mutation	Shallow Deletion	TCGA-22-4613-01
Lung Squamous Cell Carcinoma	D429Y	Missense_Mutation	Diploid	TCGA-39-5035-01
Bladder Urothelial Carcinoma	E407K	Missense_Mutation	Gain	TCGA-KQ-A41P-01
Bladder Urothelial Carcinoma	R217H	Missense_Mutation	Diploid	TCGA-2F-A9KQ-01
Bladder Urothelial Carcinoma	S278R	Missense_Mutation	Gain	TCGA-4Z-AA80-01
Bladder Urothelial Carcinoma	E396Q	Missense_Mutation	Diploid	TCGA-DK-A1AB-01
Bladder Urothelial Carcinoma	R50H	Missense_Mutation	Diploid	TCGA-DK-A3WW-01
Bladder Urothelial Carcinoma	S8L	Missense_Mutation	Gain	TCGA-XF-A9SJ-01
Hepatocellular Carcinoma	Q81L	Missense_Mutation	Gain	TCGA-G3-A5SM-01
Hepatocellular Carcinoma	S22I	Missense_Mutation	Diploid	TCGA-DD-AACY-01
Hepatocellular Carcinoma	X209_splice	Splice_acceptor_variant	Gain	TCGA-CC-A8HV-01
Hepatocellular Carcinoma	R424W	Missense_Mutation	Gain	TCGA-ED-A66Y-01
Prostate Adenocarcinoma	T33M	Missense_Mutation	Diploid	TCGA-XK-AAIW-01
Renal Clear Cell Carcinoma	L380F	Missense_Mutation	Diploid	TCGA-CJ-4869-01
Endometrial Carcinoma ***	E221K	Missense_Mutation	Diploid	TCGA-A5-A0G2-01
Uterine Endometrioid Carcinoma	R345H	Missense_Mutation	Diploid	TCGA-AJ-A3EL-01
Uterine Endometrioid Carcinoma	D257N	Missense_Mutation	Diploid	TCGA-AP-A056-01
Uterine Endometrioid Carcinoma	T266M	Missense_Mutation	Diploid	TCGA-AP-A059-01
Uterine Endometrioid Carcinoma	E198Rfs*38	Frame_Shift_Insertion	Diploid	TCGA-B5-A0JZ-01
Uterine Endometrioid Carcinoma	R217C	Missense_Mutation	Diploid	TCGA-B5-A11E-01
Uterine Endometrioid Carcinoma	E354K	Missense_Mutation	Gain	TCGA-BG-A0LX-01
Uterine Endometrioid Carcinoma	L215V	Missense_Mutation	Diploid	TCGA-BG-A18C-01
Uterine Endometrioid Carcinoma	E457K	Missense_Mutation	Diploid	TCGA-BS-A0TC-01
Uterine Endometrioid Carcinoma	E349D	Missense_Mutation	Diploid	TCGA-EO-A22R-01
Endometrial Carcinoma ***	S438 *	Nonsense_Mutation	Shallow Deletion	TCGA-EY-A1GS-01
Endometrial Carcinoma ***	L380I	Missense_Mutation	Diploid	TCGA-A5-A0G2-01
Uterine Endometrioid Carcinoma	E346 *	Nonsense_Mutation	Diploid	TCGA-A5-A2K5-01
Uterine Endometrioid Carcinoma	R310S	Missense_Mutation	Diploid	TCGA-AJ-A3EK-01
Uterine Endometrioid Carcinoma	R410=	Splice_Region, silent	Diploid	TCGA-AP-A1DV-01
Uterine Endometrioid Carcinoma	L149F	Missense_Mutation	Diploid	TCGA-B5-A3F9-01
Uterine Endometrioid Carcinoma	S325P	Missense_Mutation	Diploid	TCGA-B5-A3FC-01
Uterine Endometrioid Carcinoma	V224L	Missense_Mutation	Diploid	TCGA-EO-A3AY-01
Uterine Endometrioid Carcinoma	L131R	Missense_Mutation	Diploid	TCGA-EY-A1GD-01
Uterine Endometrioid Carcinoma	F15V	Missense_Mutation	Diploid	TCGA-FI-A2D5-01
Uterine Endometrioid Carcinoma	A247V	Missense_Mutation	Diploid	TCGA-FI-A2D5-01
Lung Adenocarcinoma	E239K	Missense_Mutation	Gain	TCGA-05-4432-01
Lung Adenocarcinoma	E225 *	Nonsense_Mutation	Gain	TCGA-44-7671-01
Lung Adenocarcinoma	D259Y	Missense_Mutation	Gain	TCGA-73-A9RS-01
Lung Adenocarcinoma	L234 *	Frame_Shift_Del	Gain	TCGA-86-A4JF-01
Lung Adenocarcinoma	Q314 *	Nonsense_Mutation	Shallow Deletion	TCGA-95-7043-01
Lung Adenocarcinoma	X209_splice	splice_acceptor_variant	Gain	TCGA-55-8301-01
Esophagogastric Adenocarcinoma	K390T	Missense_Mutation	Shallow Deletion	TCGA-L5-A4OT-01
Esophagogastric Adenocarcinoma	E288D	Missense_Mutation	Gain	TCGA-R6-A6DN-01
Cutaneous Melanoma	S325F	Missense_Mutation	ShallowDel	TCGA-EE-A2GC-06
Cutaneous Melanoma	P57R	Missense_Mutation	ShallowDel	TCGA-EE-A3JI-06
Cutaneous Melanoma	P432L	Missense_Mutation	ShallowDel	TCGA-EE-A181-06
Cutaneous Melanoma	E172K	Missense_Mutation	Diploid	TCGA-EB-A5UL-06
Cutaneous Melanoma	L326F	Missense_Mutation	Not known	TCGA-EB-A41B-01
Cutaneous Melanoma	S420F	Missense_Mutation	Diploid	TCGA-WE-A8K5-06
Cutaneous Melanoma	R186L	Missense_Mutation	Diploid	TCGA-D3-A2JB-06
Cutaneous Melanoma	R36S	Missense_Mutation	Diploid	TCGA-D3-A2JH-06
Cutaneous Melanoma	S339F	Missense_Mutation	Diploid	TCGA-D3-A8GL-06
Cutaneous Melanoma	E230K	Missense_Mutation	Gain	TCGA-D3-A8GM-06
Cutaneous Melanoma	A287T	Missense_Mutation	Diploid	TCGA-W3-A824-06
Cutaneous Melanoma	R155Q	Missense_Mutation	Diploid	TCGA-WE-A8K5-06
Diffuse Type Stomach Adenocarcinoma	R345C	Missense_Mutation	Amplification	TCGA-HU-A4GU-01
Mucinous Stomach Adenocarcinoma	R345H	Missense_Mutation	Diploid	TCGA-CD-8529-01
Intestinal Type Stomach Adenocarcinoma	T266M	Missense_Mutation	Diploid	TCGA-VQ-A91K-01
Tubular Stomach Adenocarcinoma	E349D	Missense_Mutation	Diploid	TCGA-BR-8680-01
Stomach Adenocarcinoma	V434A	Missense_Mutation	Diploid	TCGA-BR-4292-01
Intestinal Type Stomach Adenocarcinoma	R270H	Missense_Mutation	Diploid	TCGA-BR-7851-01
Stomach Adenocarcinoma	L421P	Missense_Mutation	Diploid	TCGA-BR-8372-01
Stomach Adenocarcinoma	X337_splice	splice_acceptor_variant	Diploid	TCGA-BR-8487-01
Stomach Adenocarcinoma	Y319C	Missense_Mutation	Diploid	TCGA-BR-A4QM-01
Diffuse Type Stomach Adenocarcinoma	F295S	Missense_Mutation	Diploid	TCGA-CD-A489-01
Tubular Stomach Adenocarcinoma	E407D	Missense_Mutation	Shallow Deletion	TCGA-D7-6528-01
Diffuse Type Stomach Adenocarcinoma	K390T	Missense_Mutation	Diploid	TCGA-D7-A747-01
Breast Invasive Ductal Carcinoma	R450T	Missense_Mutation	Diploid	TCGA-C8-A12T-01
Breast Invasive Ductal Carcinoma	E221K	Missense_Mutation	Diploid	TCGA-AC-A23H-01
Breast Invasive Ductal Carcinoma	Q195H	Missense_Mutation	Diploid	TCGA-A7-A26H-01
Breast Invasive Ductal Carcinoma	V77Cfs*34	Frame_Shift_Deletion	Diploid	TCGA-D8-A27V-01
Rectal Adenocarcinoma	R345C	Missense_Mutation	Diploid	TCGA-AG-3892-01
Mucinous Adenocarcinoma of the Colon and Rectum	R310C	Missense_Mutation	Diploid	TCGA-CA-6717-01
Mucinous Adenocarcinoma of the Colon and Rectum	R71W	Missense_Mutation	Diploid	TCGA-AD-5900-01
Papillary Renal Cell Carcinoma	A308G	Missense_Mutation	Diploid	TCGA-DW-7842-01
Head and Neck Squamous Cell Carcinoma	P57L	Missense_Mutation	Diploid	TCGA-CN-A640-01
Head and Neck Squamous Cell Carcinoma	R304Q	Missense_Mutation	Amplification	TCGA-UF-A71D-01
Head and Neck Squamous Cell Carcinoma	R304Q	Missense_Mutation	Gain	TCGA-CN-4727-01
Head and Neck Squamous Cell Carcinoma	D211H	Missense_Mutation	Diploid	TCGA-CR-6481-01

*: stop codon; **: Undifferentiated Pleomorphic Sarcoma/Malignant Fibrous Histiocytoma/High-Grade Spindle Cell Sarcoma; ***: Uterine Serous Carcinoma/Uterine Papillary Serous Carcinoma.
